# Thermophysical Insights into the Anti-Inflammatory Potential of Magnetic Fields

**DOI:** 10.3390/biomedicines12112534

**Published:** 2024-11-06

**Authors:** Umberto Lucia, Giulia Grisolia, Antonio Ponzetto, Thomas S. Deisboeck

**Affiliations:** 1 Dipartimento Energia “Galileo Ferraris”, Politecnico di Torino, Corso Duca degli Abruzzi 24, 10129 Torino, Italy; 2 Dipartimento di Ingegneria dell’Ambiente, del Territorio e delle Infrastrutture, Politecnico di Torino, Corso Duca degli Abruzzi 24, 10129 Torino, Italy; 3 Dipartimento di Scienze Mediche, Università di Torino, Corso Dogliotti 14, 10126 Torino, Italy; 4 Department of Radiology, Harvard-MIT Martinos Center for Biomedical Imaging, Massachusetts General Hospital and Harvard Medical School, 149 Thirteenth Street, Charlestown, MA 02129, USA

**Keywords:** inflammation, ELF-EMF, ion fluxes, thermodynamics of biosystems, biomagnetism, membrane potential

## Abstract

**Background**: Inflammation is caused by an excess of Sodium ions inside the cell. This generates a variation in the cell’s membrane electric potential, becoming a steady state from a thermodynamic viewpoint. **Methods**: This paper introduces a thermodynamic approach to inflammation based on the fundamental role of the electric potential of the cell membrane, introducing an analysis of the effect of heat transfer related to the inflammation condition. **Results**: The direct proportionality between the reduction in temperature and the increase of Na^+^ outflow may ameliorate the inflammation cascade. **Conclusions**: Based on these ion fluxes, we suggest the consideration of a ‘companion’ electromagnetic therapeutic wave concept in support of the present anti-inflammatory treatment.

## 1. Introduction

Based on experimental evidence of a magnetic field’s interaction with biological tissues in the areas of immunology [1,2,3] and oncology [4,5,6], magnetic field therapy has continuously attracted interest [7] as a potentially complementary treatment to control the inflammatory response [8,9,10]. Depending on frequency and amplitude, electromagnetic field therapies have been shown to restore equilibrium in reactive oxygen species (ROS); stabilize cytosolic Ca^2+^ [7,10], decreasing calcium-transport effects [11,12]; modulate traumatic brain injury [13]; and reduce both postoperative infections and bacterial and viral-related inflammatory responses [14,15]. Ion fluxes across the cellular membrane are essential for controlling the cell’s metabolism and state of activation; cells use genes encoding proteins to regulate membrane permeability for ions that orchestrate cell–cell communication, energy storage, and cytoskeleton assembly to address responses to environmental changes [16]. In the context of inflammation, fundamentally important ion channels include connexins, pannexins, cell–cell channels, unopposed hemichannels, and P2 receptors (P2x, P2z). Indeed, the progression of inflammation begins when the cell opens connexin and pannexin (Px1) hemichannels, followed by discharge of adenosine triphosphate (ATP) into the cell’s environment, resulting in the activation of intracellular signaling pathways concerning the activation of purinergic type 2 receptors (P2R). While at present, the activation process is unclear, the agonist tumor necrosis factor receptors TNFR-1 and TNFR-2 are thought to be responsible for the cell’s membrane potential increase related to the Na^+^ channel [17]. Indeed, ion transport across cell membranes generates membrane potentials (Na^+^, K^+^ and Cl^−^ transport) and provides the osmotic gradients (Na^+^ and Cl^−^ fluid transport) required for transmembranous and paracellular fluid transport [18]. This process of membrane potential increase alone can explain the neuropathic effects related to inflammation.

Inflammation represents the primary response of the immune system to infection or tissue injury [19]. Generally, an inflammatory process starts with the cell’s membrane potential increasing; a process mediated by the Na^+^-channel [17]. The main passive transport influx of Na^+^ is the ENaC (Epithelial Sodium Channel), while Na^+^/K^+^-ATPase facilitates the main active transport outflow of Na^+^ and inflow of K^+^. In the inflammation state, sensory transduction of pain [20] has been shown to depend on voltage-gated Na^+^, Ca^2+^, and K^+^ channels, ligand-gated ion channels, purinergic receptors, and transient receptor potential channels [21]. Moreover, in macrophages, high salt leads to a Na^+^/Ca^2+^-exchanger 1 (NCX1)-dependent increase in intracellular Na+ levels [22], highlighting a strict relation between the Na^+^ and Ca^2+^ fluxes. Furthermore, some Ca^2+^ channels mediate neurotransmitter release and Ca^2+^-dependent enzyme activation [23]. Thus, ion channels and transporters control the intracellular calcium concentrations [24] and the endosomal pH [25], which impact the immune system response. Indeed, the rise in the intracellular calcium concentration generated by SOCE (Store-Operated Ca^2+^ Entry) [24] is related to downstream B- and T-cell receptors (BcR and TcR), enhanced by the transient receptor potential cation channel TRPM7, in turn influenced by variations in Mg^2+^ concentrations or the Fc receptors pathways [26]. Modification of the permeability of calcium channels or transporters can be induced by several different mechanisms [27]:

- Engagement of Receptor-Operated Calcium Entry (ROCE), modulated by auto- or paracrine adenosine triphosphate (ATP), adenosine diphosphate ribose (ADPR), and multiple other chemical ligands or physical stimuli. In this context, the inositol-phosphate 3 (IP3) receptor channel, which allows calcium fluxes into the cytoplasm, is activated by phospholipases and is paired with the production of diacylglycerol (DAG), which is a ligand for some receptors and channels. Intracellular phospholipases, modulated by magnesium or zinc interchanges, determine signal cascades downstream of the B- and T-cell receptor (BcR and TcR);
- Premature release of calcium from Store-Operated Ca^2+^ Entry (SOCE);
- Variations in Voltage-Operated Ca^2+^ Entry (VOCE [20]), and
- Variations in Na^+^ driving effects.

This biochemical evidence points to a fundamental role of ion transfer in the process of inflammation, and it also emphasizes a concomitant variation of the cell’s membrane potential. Specifically, the role of Na^+^ and Ca^2+^ is thought to be essential for the control of inflammation—Na^+^ in particular, because this ion is the cause of the beginning of the inflammatory state. To summarize, the aforementioned biomedical evidence allows us to state the following:

- Energy processes involve mitochondria activities; as such, the control of energy fluxes must be considered as a pillar of any approach to inflammation;
- Na^+^ flux is at the inception of inflammation; as such, a decrease in Na^+^ inner concentration can represent a fundamental therapeutic strategy to more effectively manage if not reduce inflammation. In this context, the role of Ca^2+^ appears to be important.

In this paper, we therefore analyze such ion transport based on irreversible thermodynamics. We propose a novel concept for anti-inflammation therapy by controlling ion fluxes, and consequently, electromagnetic therapy is suggested as an adjuvant tool to improve current, conventional treatment modalities. To do so, Section 2 describes the thermodynamic approach, while Section 3 summarizes the results. The last section discusses potential biomedical implications. As no experimental support is provided, the analysis developed here is theoretical only; however, it represents a first thermodynamic approach to the analysis of inflammation.

## 2. Methods

The cell membrane presents an electric potential difference, Δϕ, generated by the concentration of different ions (Na^+^, K^+^, Cl^−^, Ca^2+^, etc.): Δϕ is measured between the cytoplasm and the extracellular environment in relation to the environment [28]; consequently, it assumes a negative value. The membrane’s electric potential is theoretically described by the Goldman–Hodgkin–Katz equation [29,30,31,32]:

(1) $$\Delta \phi =\frac{RT}{F}\;\log_{10}\left(\frac{P_{{\mathrm{Na}}^{+}}{\left[{\mathrm{Na}}^{+}\right]}_{\mathrm{outside}}+P_{K^{+}}{\left[K^{+}\right]}_{\mathrm{outside}}+P_{{\mathrm{Cl}}^{-}}{\left[{\mathrm{Cl}}^{-}\right]}_{\mathrm{outside}}}{P_{{\mathrm{Na}}^{+}}{\left[{\mathrm{Na}}^{+}\right]}_{\mathrm{inside}}+P_{K^{+}}[K_{\mathrm{inside}}^{]}+P_{{\mathrm{Cl}}^{-}}{\left[{\mathrm{Cl}}^{-}\right]}_{\mathrm{inside}}}\right)$$

where [A] is the concentration of ion A in mol m^−3^, R=8.314 J mol^−1^K^−1^ is the universal constant of ideal gasses, *T* stands for the absolute temperature, *F* is the Faraday constant, and *P* denotes the relative permeability, such that $P_{{\mathrm{Na}}^{+}}=0.04$, $P_{K^{+}}=1$, and $P_{{\mathrm{Cl}}^{-}}=0.45$. The concentration, chemical potential, and electric membrane potential of some ions, in normal cells, are reported in Table 1.

Regarding inflammation and the definition of a cell’s membrane potential, the increase in sodium potential can be obtained by Na^+^ inflow into the cell. This Na^+^ flux determines the electric work corresponding to the power requirement:

This Na^+^ flux determines a membrane potential variation corresponding to the power requirement:

(2) $${\dot{W}}_{el,{\mathrm{Na}}^{+}}=J_{{\mathrm{Na}}^{+}}\cdot E\;\;4\pi {\langle R\rangle }^{2}\;d_{memb}$$

where J=|J| is the flux density [A m^−2^], E=|E| depicts the electric field, $\langle R\rangle$ is the mean value of the cell’s radius, and $d_{memb}$ stands for the cell membrane depth. The electric field at the cell membrane can be evaluated considering that the electric potential at a normal cell’s membrane is approx. –70 mV. The thickness of the membrane is on the order of 0.004 μm, so the electric field of the cell membrane can be approximated as $-1.75\times 10^{7}$ V m^−1^.

Now, we consider the first law of thermodynamics [34]:

(3) $$\dot{Q}-{\dot{W}}_{el,{\mathrm{Na}}^{+}}=\frac{dU}{dt}$$

where $\dot{Q}$ represents the heat power, U=mcT is the internal energy, m=ρV is the mass of cell, ρ is the cell density, *V* is the cell volume, *c* is its specific heat, and *T* stands for temperature. In the sodium inflow process, the heat power is null. Consequently:

(4) $$\rho \;c\;\frac{dT}{dt}\;V=J_{{\mathrm{Na}}^{+}}\cdot E\;\;4\pi {\langle R\rangle }^{2}\;d_{memb}$$

where $\rho \approx 10^{3}$ kg m^−3^ denotes the cell density, and c≈4186 J kg^−1^ K^−1^ is the specific heat of the cell.

**Figure 1:**
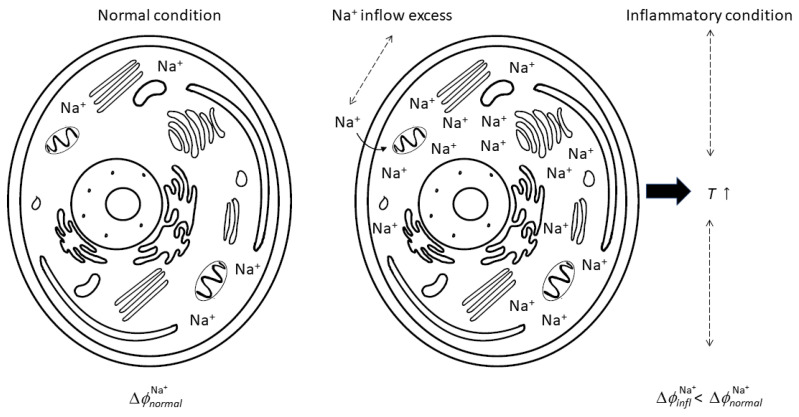
The process of inflammation, from sodium inflow excess to inflammation inception that determines the membrane’s electric potential variation, with a consequent stationary state of inflammation.

Hyperpolarization (see Figure 1) determines the activation of the Ca^2+^-K^+^ channel [35,36], with the consequence that the Ca^2+^-K^+^ channel emerges as a fundamental control lever of the membrane’s electric potential. In this context, water influx has been shown to be critical as well [35,37].

Next, proteins play a fundamental role in ion transport; those in the cytosol can be modified with regard to their functions by phosphorylation or dephosphorylation. An ion actively crosses the membrane against its electrochemical potential, whereby the necessary energy is derived either from the hydrolysis of ATP or from the movement of a co-transported or coupled ion along its electrochemical gradient. In this context, the role played by the H^+^-ATPase is essential, because it moves positive charges into the cell while generating a large membrane voltage as well as a pH gradient [38,39,40,41]. Protein phosphorylation is an important cellular regulatory mechanism because many enzymes and receptors [42,43,44] are activated or deactivated by phosphorylation [45,46,47,48].

This implies the activation of ion fluxes of potassium, chlorine and calcium by Na^+^-K^+^-ATPase, cAMP, and CFTR:

(5) $$\frac{dU}{dt}=-\nabla \cdot J\;\;V=-V\;\sum_{{\mathrm{Cl}}^{-}{,K}^{+}{,\mathrm{Ca}}^{2+}}\nabla \cdot J_{{\mathrm{Cl}}^{-}{,K}^{+}{,\mathrm{Ca}}^{2+}}$$

with a related variation in the cell’s membrane electric potential:

(6) $$\Delta \phi_{{\mathrm{Na}}^{+}}=\Delta \phi_{{\mathrm{Cl}}^{-}}+\Delta \phi_{K^{+}}+\Delta \phi_{{\mathrm{Ca}}^{2+}}$$

Moreover, Equation (4) states the direct proportionality between the increase in temperature, i.e., dT/dt>0, and the increase in Na^+^ ions inside the cell. Thus, a cell must export heat power to decrease its temperature:

(7) $$\delta \dot{Q}=-\alpha \;\left(T-T_{0}\right)\;dA$$

where α≈0.023Re0.8Pr0.35λ/〈R〉 denotes the coefficient of convection, with λ≈0.56 W m^−1^K^−1^ denoting conductivity, Re≈0.2 denoting the Reynolds number, and Pr≈0.7 denoting the Prandtl number [49]. *A* is the area of the cell membrane, *V* stands for the cell volume, and 〈R〉=dV/dA≈V/A is the mean radius of the cell.

## 3. Results

Our analysis focuses on the thermophysical consequences of the aforementioned ion fluxes in inflammation processes. In particular, we determine how ion fluxes induce the need for heat transfer and restoration of the healthy cell’s membrane electric potential by inducing new ion fluxes. Indeed, considering Equation (7), we can see that if an increase in heat outflow is induced, a Na^+^ outflow follows, as expressed in Equations (2) and (4):

(8) $$\dot{Q}=-\alpha \;\left(T-T_{0}\right)\;A=\frac{dU}{dt}=\rho \;c\;\frac{dT}{dt}\;V=J_{{\mathrm{Na}}^{+}}\cdot E\;\;4\pi {\langle R\rangle }^{2}\;d_{memb}$$

which implies:

(9) $$\delta \dot{Q}<0\Rightarrow J_{{\mathrm{Na}}^{+}}<0$$

The approach developed here highlights, for the case of inflammation, the strict relation between the temperature increase as a consequence of an excess of Na^+^ inflow. Consequently, in such an inflamed milieu, a cell generates a change in ion fluxes, i.e., Cl^−^, K^+^, and Ca^2+^, and thermal outflow.

Furthermore, such ion fluxes then emphasize the need for membrane potential control, with all the biophysical consequences for cell behavior. Now, considering that the variation in the electric membrane potential ϕ can be expressed as [33]:

(10) $$nF\;d\phi =dH-T_{0}\;dS-2.3\;n\;RT_{0}\;d\left(\mathrm{pH}\right)=dH-\delta Q-2.3\;n\;RT_{0}\;d\left(\mathrm{pH}\right)$$

where *n* is the number of moles of ion charges considered, dH is the the enthalpy variation, $T_{0}$ is the environmental temperature of the cell, dS is the the entropy variation, *R* and *F* are the universal ideal gas and the Faraday constants, respectively, and *d*pH is the variation in the pH between the two membrane surfaces. While the variation in the membrane electric potential is caused by the Na^+^ and K^+^ fluxes, the membrane voltage regulation, however, is controlled by the Cl^−^ flux, so changes in membrane voltage cause a flux of Cl^−^ to restore normal conditions.

Now, in keeping with our goal to see if electromagnetic field therapies may be able to support current conventional medical strategies to combat inflammation, and considering Equations (6) and (10), it follows:

(11) $$n_{{\mathrm{Na}}^{+}}\;F\;d\phi -dH_{{\mathrm{Na}}^{+}}+2.3\;n_{{\mathrm{Na}}^{+}}\;RT_{0}d{\left(\mathrm{pH}\right)}_{{\mathrm{Na}}^{+}}=-\delta Q_{infl}$$

That is, under normal conditions, the temperature difference $T-T_{0}$ remains constant, but during inflammation, it increases, so this ‘extra’ outflow of heat $-Q_{infl}$ is required to alleviate inflammation and restore ‘normal’ conditions. This last relation highlights the link between the membrane’s electric potential and cross-membrane heat transport, which emphasizes the control of the cell’s energy generation, i.e., the mitochondria activity. From this last equation, it is possible to evaluate the fluxes of Na^+^ ions that generate inflammation:

(12) $$\Delta n_{{\mathrm{Na}}^{+}}=\frac{\rho \;c\;(T_{high}-T)\;V}{F\;\Delta \phi_{{\mathrm{Na}}^{+}}-\Delta h_{{\mathrm{Na}}^{+}}+2.3\;RT\;\Delta {\left(\mathrm{pH}\right)}_{{\mathrm{Na}}^{+}}}$$

where T=37.4 °C is the temperature of the cell under normal conditions, and $T_{0}=37.0$ °C the cell’s environmental temperature.

**Figure 2:**
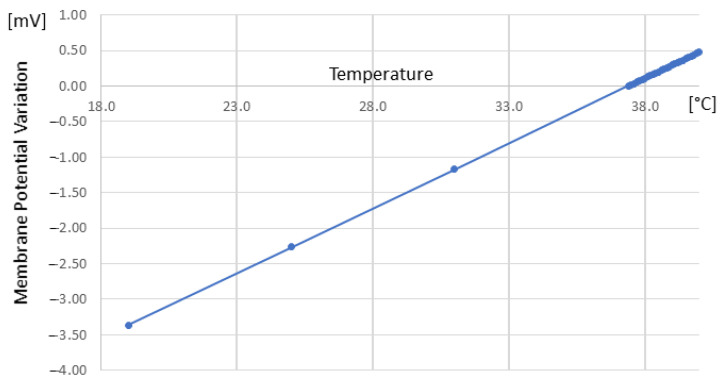
Membrane electric potential variation in relation to temperature variation, evaluated using Equations (7) and (10).

The Na^+^ variation determines a change in the membrane’s electric potential. Moreover, temperature determines changes in the heat flux, so we expect that a change in sodium flux occurs as well. Experimentally, this means that during inflammation, temperature variation must determine changes in the electric membrane potential. We have numerically simulated this effect for a range in temperature [19–40] °C, as represented in Figure 2. As stated in the Introduction section, while we have not yet performed experimental works ourselves, in comparing our results with experimental data reported in literature, we find good agreement: e.g., at 19 °C, a decrease in potential of 3.36 mV is reported (compared with the results of ref. [50] of (2.0±2.1) mV), while for 25 °C, a decrease in the potential of 2.27 mV is reported (compared with the results of ref. [50] of (1.6±2.2) mV) and at 31 °C, a decrease of potential of 1.17 mV is reported (compared with the results of ref. [50] of (0.8±2.0) mV).

Our results are also confirmed in ref. [51], which reports that under complete Freund’s adjuvant (CFA)-induced peripheral inflammation, the electric membrane potential changes from (−37.7±1.5) mV in the control to (−42.5±1.1) mV under inflammation.

Now, to introduce a possible thermodynamic approach for an electromagnetic therapy to reduce inflammation, we consider that electromagnetic waves generate a radiation pressure [52]:

(13) $$p=\frac{\varepsilon \;E^{2}}{2}=\frac{B^{2}}{2\;\mu }$$

where *E* and *B* are the amplitudes of the electric field and magnetic field components, respectively, μ is the magnetic permeability, and $c=1/\sqrt{\mu_{0}\varepsilon_{0}}\approx 3\times 10^{8}$ m s^−1^ is the velocity of light, with $\varepsilon_{0}=8.854\times 10^{-12}$ A s N^−1^m^−1^ being the electric permittivity and $\mu_{0}=4\pi \times 10^{-7}$ H m^−1^ being the magnetic permeability in a vacuum, respectively. As a consequence of this pressure, the membrane is subjected to an elastic force [53]:

(14) $$F=\frac{\varepsilon \;E^{2}}{2}\;A\;2\pi \;r$$

where *A* is the surface of the membrane that the electromagnetic wave hits, while *r* represents the mean value of the internal cell radius. Due to the membrane surface deformation caused by this force, the membrane’s electric potential is affected by local variation:

(15) $$\Delta \phi =E\;A=\sqrt{\frac{F_{el}\;A}{\pi \;\varepsilon \;r}}$$

forcing the ion channels to open for inflows and outflows. As such, since in our quest to mitigate inflammation, we need to force Na^+^ outflow only, we must find the values of *E* and *B*.

To show a use case example of this approach, we consider nano-mechanical responses to elastic perturbations [53,54]. Therefore, concerning, e.g., osteocytes, we can summarize the following properties:

- Diameter in the range of 20–100 µm
- Membrane depth in the range of 5–10 nm

To validate the current model, we start with the experimental results obtained in ref. [55]. Considering the previous radius, we can obtain the range of frequencies useful to reduce inflammation, i.e., (13.5±1.4) Hz for 20 µm with its harmonic frequencies and (0.6±0.1) Hz for 100 µm with its harmonic frequencies. In particular, some harmonic frequencies are in the ranges of (1.9±0.2) Hz, (4.5±0.5) Hz, (13.5±1.4) Hz, (26.9±2.7) Hz, (53.8±5.4) Hz, (80.7±8.1) Hz, etc. The experimental findings in ref. [55] reveal the following:

- 2 Hz affects inflammation by downregulating TNF-α and IL-1β
- 4 Hz reduces oxidative stress;
- 12 Hz improves local microcirculation;
- 15 Hz increases alkaline phosphatase activity (ALP) and chondrogenesis;
- 30 Hz affects inflammation by downregulating IL-10;
- 50 Hz impacts inflammation by reducing chemokine production;
- 75 Hz upregulates A2A and A3 adenosine receptors and induces anti-inflammatory effects.

These frequencies are in agreement with the ones obtained using our model. Another confirmation of our thermodynamic approach is the evaluation of Fe^2+^ fluxes developed in ref. [56], whose results concur with the values of iron accepted in medicine.

## 4. Discussion and Conclusions

This paper introduces a thermodynamic approach to inflammation based on the fundamental role of the cell membrane’s electric potential. It highlights how heat transfer and ion transport allow the cell to control the membrane’s electric potential. Moreover, we suggest a ’companion’ electromagnetic therapy in support of the present anti-inflammatory treatment by proposing a possible mechanism of action, which up until now has not been fully understood. Supporting our conjecture, we cite ref. [57], which used conducting polymer microwires to control the resting membrane potential of *Escherichia Coli* cells, with the aim of providing a new, non-invasive, cellular-scale tool to control this membrane potential with a high spatial precision. This paper demonstrates how the ability of controlling the membrane potential allows us to induce cells to pump out ions in response to changes in polarization. As such, we believe that accelerating Na^+^-outflow to curtail inflammation, as proposed here, may eventually become technically feasible. Next, experimental data confirming the effects of electromagnetic fields are summarized in Table 2; some of them refer to clinical studies such as ref. [7] which, for instance, reported a reduction in inflammation in osteoarthritis related to the use of a 1.5 mT magnetic field (75 Hz). Table 2 highlights immunological studies that clearly demonstrate that low-frequency magnetic field therapy effectively interacts with cells and tissues. This therapeutic modality may therefore present not only a viable alternative but also a powerful complementary approach to existing treatments, delivering a faster reduction of the inflammatory response. The growing interest in magnetic field therapy is well-founded, as published research strongly indicates that this non-invasive and cost-effective method may surpass the safety of drugs and surgical procedures in reducing inflammation [55,58,59].

In a literature review, ref. [62], ELF-EMF therapy (ELF—extreme low frequency; EMF—electromagnetic field) in orthopaedic practice was examined for its ability to enhance tissue repair and growth, in addition to both a chemical approach [63,64,65] and a possible alternative therapeutic approach. Pre-clinical studies have indicated that biophysical stimuli interact with cell membranes, hence agreeing with our findings. This therapy has been shown to increase the proliferation, synthesis, and release of growth factors in bone tissue. In particular cells, EMFs have been found to have anti-inflammatory and chondroprotective effects. In animal studies, this treatment nearly doubled the mineralization rate of newly formed bone, inhibited the progression of osteoarthritic cartilage degeneration, and preserved cartilage quality. Biophysical stimulation has been successfully used in clinical settings to promote the healing of fractures and non-unions, as well as to improve joint function and reduce inflammation in periarticular tissues. The effects of pulsed radiofrequency electromagnetic fields (PRF-EMFs) exposure were studied in ref. [66] on the inflammatory, antioxidant, cell proliferation, and wound healing characteristics of human primary dermal fibroblasts collected from patients with venous leg ulcers. The results emphasized the ability of PRF-EMFs to modulate the TGFβ, COX2, IL6, IL1β, and TNFα gene expression in exposed ulcers. This reduced the related inflammation and confirmed that exposure to PRF-EMFs can represent a strategy to help tissue repair, regulating mediators involved in the wound healing process. Concerning the effects of EMF on bone and joint formation, maintenance, and regeneration, the results reviewed in ref. [67] point out that EMFs stimulate chondrocyte proliferation, differentiation, and extracellular matrix synthesis via the release of anabolic morphogens such as bone morphogenetic proteins and anti-inflammatory cytokines by adenosine receptors A2A and A3 in both in vitro and in vivo investigations.

In summary, our thermodynamic approach to inflammation modeling highlights the pivotal role of the cell membrane’s electric potential as well as the direct correlation between temperature elevation and the increase in Na^+^ ions within the cell. This suggests that therapeutically facilitating Na^+^ outflow could potentially alleviate the inflammatory burden. Cautiously extrapolated, as a result of these ion exchanges, we propose considering an accompanying electromagnetic therapeutic wave concept to bolster current anti-inflammatory interventions.

## Figures and Tables

**Table 1:** Concentration, chemical potential (in water solution), and electric membrane potential of some ions in normal cells [33].

Ion	Extracellular	Intracellular	Chemical	Membrane
Species	Concentration	Concentration	Potential $\mu_{i}$	Potential $E_{i}$
	$\times 10^{-3}$ [M]	$\times 10^{-3}$ [M]	$\times 10^{3}$ [J mol^−1^]	$\times 10^{-3}$ [V]
Na^+^	18	150	−261.89	+56
K^+^	140	5	−283.26	−89
Cl^−^	120	7	−131.26	−76
Ca^2+^	1.2	0.1	−553.04	+125

**Table 2:** Examples of evidence of possible use of ELF-EMF in medicine from the literature [7].

Disease	Frequency [Hz]	Key Finding	Reference
Arthritis	60	Reduction in pain and inflammation	[8]
Back pain	64	Statistically significant for reducing pain	[60]
Carpal tunnel	20	Statistically significant pain reduction *	[61]

* short- and long-term.

## Data Availability

All the references for the data are cited in the paper.
